# A lethal disease model for New World hantaviruses using immunosuppressed Syrian hamsters

**DOI:** 10.1371/journal.pntd.0006042

**Published:** 2017-10-27

**Authors:** Valentijn Vergote, Lies Laenen, Bert Vanmechelen, Marc Van Ranst, Erik Verbeken, Jay W. Hooper, Piet Maes

**Affiliations:** 1 KU Leuven–University of Leuven, Department of Microbiology and Immunology, Laboratory of Clinical Virology, Zoonotic Infectious Diseases unit, Leuven, Belgium; 2 KU Leuven–University of Leuven, Department of Imaging & Pathology, Translational Cell and Tissue Research, Leuven, Belgium; 3 Department of Molecular Virology, Virology Division, United States Army Medical Research Institute of Infectious Diseases, Fort Detrick, MD, United States; NIAID Integrated Research Facility, UNITED STATES

## Abstract

**Background:**

Hantavirus, the hemorrhagic causative agent of two clinical diseases, is found worldwide with variation in severity, incidence and mortality. The most lethal hantaviruses are found on the American continent where the most prevalent viruses like Andes virus and Sin Nombre virus are known to cause hantavirus pulmonary syndrome. New World hantavirus infection of immunocompetent hamsters results in an asymptomatic infection except for Andes virus and Maporal virus; the only hantaviruses causing a lethal disease in immunocompetent Syrian hamsters mimicking hantavirus pulmonary syndrome in humans.

**Methodology/Principal findings:**

Hamsters, immunosuppressed with dexamethasone and cyclophosphamide, were infected intramuscularly with different New World hantavirus strains (Bayou virus, Black Creek Canal virus, Caño Delgadito virus, Choclo virus, Laguna Negra virus, and Maporal virus). In the present study, we show that immunosuppression of hamsters followed by infection with a New World hantavirus results in an acute disease that precisely mimics both hantavirus disease in humans and Andes virus infection of hamsters.

**Conclusions/ Significance:**

Infected hamsters showed specific clinical signs of disease and moreover, histological analysis of lung tissue showed signs of pulmonary edema and inflammation within alveolar septa. In this study, we were able to infect immunosuppressed hamsters with different New World hantaviruses reaching a lethal outcome with signs of disease mimicking human disease.

## Author summary

Hemorrhagic fever viruses have become of much greater importance in recent years. The more prominent presence of these viruses in a day-to-day setting and their impact on global health has had a positive impact on studying these viruses. Resolving their disease mechanisms and ways to intervene in their replication and associated health burden will stay a challenge for the upcoming decades. This challenge also counts for hantavirus, an emerging zoonosis. No vaccine or antiviral treatment is WHO prequalified or has shown to elicit a good protective or therapeutic antiviral response in humans. One of the main reasons is the lack of good animal disease models and the research focusing on this topic. Without a representative model, it is difficult to investigate new potential drugs. Within this setting we focused on establishing a disease model for the most prominent New World hantaviruses.

## Introduction

Hantaviruses are part of the *Hantaviridae*, a virus family within the order *Bunyavirales* in which the genus *Orthohantavirus* harbors 41 species. These viruses are enveloped, negative sense single-stranded RNA viruses that are not transmitted by arthropods as other families within the order *Bunyavirales* but by rodents and insectivores. Hantavirus is a segmented virus consisting of 3 segments; S segment, M segment and L segment. The segments encode the nucleocapsid, the glycoproteins (Gn and Gc) and the RNA-dependent RNA polymerase, respectively.

Rodents are the natural hosts for hantaviruses and the primary source for human infections, although new hantaviruses were recently discovered or isolated from bats, moles and shrews[1–3]. Evidence for transmission and infection between these newly discovered viruses and their natural hosts and humans is not clear and should be investigated further.

Hantaviruses have a clinical impact as they are the source for two diseases; hemorrhagic fever with renal syndrome (HFRS) in the Old World/Eurasia and hantavirus pulmonary syndrome (HPS) in the New World/the Americas. In Western Europe, Puumala virus is responsible for a, mostly milder form of HFRS, named nephropathia epidemica. The complexity and the presence of overlapping symptoms seen in illness caused by several distinct hantaviruses raised the idea of ‘hantavirus disease’ as a more general name [4].

HPS onset is characterized by typical viral symptoms: headache, fever, myalgia, nausea, diarrhea and even abdominal pain are frequently present. This onset of disease is called the prodromal phase and is followed by the cardiopulmonary phase, the diuretic phase and the convalescent phase. The second phase starts rather abrupt and mortality is mostly seen within 24 hours after the onset of respiratory symptoms (pulmonary edema, respiratory distress, shock, cardiac failure). When patients survive this phase the diuretic phase starts, which is characterized by high urine flow rates and improvement of the respiratory symptoms. After entering this phase patients are believed to have a relatively good prognosis of survival. The convalescent phase following diuresis is the start of recovery, which can take several months and even years [5–7].

Since its discovery in 1978, [8] hantavirus, an emerging zoonotic virus with a worldwide distribution, has been the subject of various scientific research projects across multiple domains. One such domain is the search for good and effective antiviral products and vaccines. However, when looked at literature the success of hantavirus antivirals is limited [9–12]. One of the major problems is the lack of an animal disease model for hantaviruses. Several attempts have been made to develop a sustainable model but only one model has shown to be really scientifically usable, a lethal disease model for HPS [13]. In this model, immunocompetent golden Syrian hamsters were infected with Andes virus (ANDV). Starting 11 days post infection, hamsters died or showed signs of respiratory distress and a human-like disease pattern [13]. Surprisingly, only ANDV was potentially lethal for hamsters whereas other New World hantaviruses showed no such outcomes.

Building further on this model, the use of immunosuppressive agents was tested by Brocato et al. with the goal of adapting the model for Sin Nombre virus (SNV) [14]. SNV was previously tested in immunocompetent hamsters but did not cause any disease[13].

The scope of the current study is to further explore the use of immunosuppressive treatment to achieve an animal disease model for New World hantaviruses. Several New World hantaviruses were tested, based on the results from the model adaptation by Brocato et al., who saw the development of an HPS-like disease when using immunosuppressed Syrian hamsters to study.

## Materials and methods

### Ethics statement

All animal work was done in a biosafety level 3+ setting. This study was approved and supervised by the KU Leuven Animal Welfare Body (KU Leuven 206/2012—LA1210186) in compliance with Belgian and European statutes and regulations relating to animals and experiments involving animals.

### Syrian golden hamsters

Female Syrian golden hamsters, with a minimum weight of 120 g, were purchased from Janvier Labs (Saint Berthevin, France). All hamsters were housed per pair in an individually ventilated cage (IVC) system provided with high absorbent nesting material. Food and water were available *ad libitum*. The animals were maintained in an approved biosafety level 3+ facility under conditions that met all requirements for animal care. Animals were sacrificed by lethal pentobarbital injection upon sedation with 2.5% isoflurane.

### Viruses and virus challenge

Different hantaviruses were used to challenge hamsters: Andes virus strain Chile-9717869, Bayou virus [15], Black Creek Canal virus [16], Caño Delgadito virus strain VHV-574, Choclo virus strain 588, Laguna Negra virus strain 510B, Maporal virus strain HV9021050 (kindly provided by Dr. Robert Tesh, World Reference Center for Emerging Viruses and Arboviruses, University of Texas Medical Branch, Galveston, TX, USA), Puumala virus strain CG1820, and Sin Nombre virus strain Convict Creek HN107. These viruses were brought in culture and passaged on Vero E6 cells (Vero C1008, CRL-1586; ATCC). Dulbecco's Modified Eagle Medium (DMEM, Gibco, Thermo Fisher Scientific, Erembodegem, Belgium) enriched with 10% fetal bovine serum (Thermo Fisher Scientific) and 1% sodium bicarbonate (Thermo Fisher Scientific) was used to culture the cells in T75 cell culture flasks. Cells were kept in a humidified CO_2_ incubator (5%) on 37°C. Ten days post infection, cells were disrupted by 3 consecutive freeze/thaw cycles followed by sucrose cushion ultracentrifugation to purify virus preparations. Viral stocks were titrated by plaque assay. Female Syrian hamsters were, upon sedation with isoflurane (IMPAC 6 veterinary anesthesia machine), subjected to an intramuscular injection of 200 μL virus suspension (2,000 PFU). Sterile PBS was used to dilute the virus. Hamsters were monitored continuously during the entire procedure and upon recovery rehoused in the IVC system.

### Focus forming plaque assays

Hantavirus focus forming plaque assays were performed as described elsewhere [17]. Briefly, virus dilutions were serially made and inoculated onto 6-well plates containing confluent Vero E6 cell monolayers. After adsorption for 1h the wells were overlaid with a mixture of agarose (1%) and basal Eagle’s medium (Thermo Fisher Scientific) and plates were incubated for 10 days. Virus-infected cells were detected with hantavirus-specific rabbit polyclonal antisera (kindly provided by Prof. D.H. Krüger, Institut für Virologie, Charité, Berlin, Germany), followed by peroxidase-labeled goat anti-rabbit antibodies and substrate.

### Dexamethasone and cyclophosphamide treatment

Hamsters were treated for 11 days with dexamethasone (Rapidexon 2 mg/ml, Eurovet Animal Health BV, The Netherlands) and cyclophosphamide (cyclophosphamide monohydrate, Sigma Aldrich, Belgium) starting 3 days prior to virus infection and ending 7 days post infection using 25G x5/8 needles (BD, Belgium). The used dosing scheme was composed of daily administrations of dexamethasone and cyclophosphamide based on the dosing scheme described by Brocato *et al* [14]. Boost doses given the days prior to infection consisted of 140 mg/kg cyclophosphamide and 16 mg/kg dexamethasone 3 days prior to infection, 2 days before infection 8 mg/kg dexamethasone was given and 1 day prior to infection 8 mg/kg dexamethasone and 100 mg/kg cyclophosphamide were administered intraperitoneally. On the day of virus challenge 4 mg/kg dexamethasone was given to the hamsters followed by a combination of 4 mg/kg dexamethasone and cyclophosphamide on day 1. A cyclic pattern as described in Table 1 was used for the further proceedings of the experiment.

**Table 1:** Dexamethasone and cyclophosphamide dosing scheme.

	Pre-infection dosing			infection	Maintenance dosing						
**Days (relative to date of infection)**	**-3**	**-2**	**-1**	**0**	**1**	**2**	**3**	**4**	**5**	**6**	**7**
**Dexamethasone (mg/kg)**	16	8	8	4	4	4	4	4	4	4	4
**Cyclophosphamide (mg/kg)**	140	NT	100	NT	100	NT	NT	100	NT	NT	100

NT: Not Treated

### Passive immunization

Hamsters were anesthetized as described above, and 10,000 neutralizing antibody units of convalescent heat-inactivated serum from C57BL/6 mice (Provided by the CEV Zoonotic Infectious Diseases Unit, KU Leuven, Belgium) diluted in sterile PBS (pH 7.4), were injected subcutaneously with a 1-ml syringe with a 25-gauge needle one day before infection with Bayou virus (BAYV) or Black Creek Canal virus (BCCV) [18].

### RNA isolation, RT-PCR and sequencing

Organs were extracted using the RNeasy mini kit (Qiagen) according to the manufacturer’s instructions. The tissue homogenizing step was performed on a Minilys homogenizer (precellys, Bertin) using zirconium oxide beads (2.8 mm; Precellys, Bertin). RT-PCR was performed using strain specific primers for BCCV and BAYV (HANTA-S-BCCV-F: 5’ CAC ACT ACA GAA CAG ACG G 3’; HANTA-S-BCCV-R: 5’ CTG CCT TCC TCG TGT TGA C 3’; HANTA-S-BAYV-F: 5’ TTC CCA GCC CAA ATC AAG GC 3’; HANTA-S-BAYV-R: 5’ GGA AGT AAG CAC CAT TTG TCG A 3’) targeting the S-segment of these viruses. The One step RT-PCR kit (Qiagen) was used to perform the reaction according to the manufacturer’s instructions. Positive bands were confirmed with chain-terminating sequencing as previously described [19].

### Histology

Lung tissue samples were fixed in 10% buffered formalin and embedded in paraffin. Tissue sections (4 μm) were stained with hematoxylin and eosin for routine histological examination. Histopathological grading of lung tissue was performed using a standardized scoring system between 0 (absent), 0.5 (minimal/spotty), 1 (few spots), 2 (multifocal areas), and 3 (diffuse confluent areas) [20].

### Statistical analysis

Kaplan-Meier survival curves were made and illustrated using GraphPad Prism 6. The significance threshold (*P* value) was set at 0.05. *P* values were calculated by both a log-rank test and a Gehan-Breslow-Wilcoxon test for all groups.

## Results

### Infection of immunosuppressed hamsters with New World hantaviruses results in high mortality

In order to develop a disease model for New World hantaviruses, 10 groups of 8 Syrian gold hamsters were treated with a dexamethasone/cyclophosphamide combination (DEX/CYP) according to the treatment scheme in Table 1. Nine of these groups were challenged with 2,000 PFU of a specific hantavirus on day 0. Eight New World hantaviruses were tested of which Andes virus (ANDV) and Sin Nombre virus (SNV) served as positive controls. A European strain, Puumala virus (PUUV), was also included in the study. A last group only received immune suppression and served as a negative control. Daily, hamsters were weighed and checked for the presence of signs of disease. All virus-treated hamsters showed significant weight loss (S1 Table).

**Fig 1:**
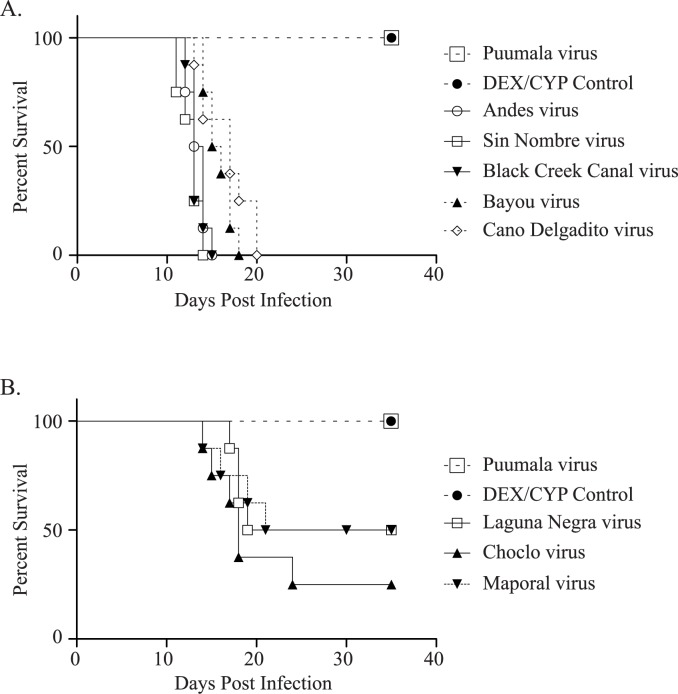
Survival curves of golden Syrian hamsters infected with New World hantaviruses. A) Infection with New World hantaviruses (BAYV, BCCV, CDV, SNV and ANDV) leading to a mortality rate of 100%. B) Infection with New World hantaviruses leading to a mortality rate of 75% for CHOV and 50% for LNV and MAPV. BAYV, CDV, BCCV, ANDV and SNV, Log-rank: p value <0.0001, Gehan-Breslow-Wilcoxon: p value = 0.0002; LNV and MAPV, Log-rank: p value = 0.0251, Gehan-Breslow-Wilcoxon: p value = 0.0265; and CHOV; Log-rank: p value = 0.0024, Gehan-Breslow-Wilcoxon: p value = 0.0032.

The onset of disease was abrupt and mortality occurred within 24–36 hours after the first signs. For ANDV, 100% mortality was observed between 12 and 15 days post infection (dpi), for Bayou virus (BAYV) between 14 and 18 dpi, for Black Creek Canal virus (BCCV) between 12 and 15 dpi, for Caño Delgadito virus (CDV) between 13 and 20 dpi and for SNV between 11 and 14 dpi. For Choclo virus (CHOV), Laguna Negra virus (LNV), and Maporal virus (MAPV), respectively 75%, 50% and 50% mortality was observed 35 days post infection, with onset of mortality starting 14 dpi for CHOV and MAPV and 17 dpi for LNV. All hamsters of the negative control group (treated with DEX/CYP, but not infected) and the PUUV group were still alive at the termination of the experiment 35 days post infection with no noted signs of disease (Fig 1).

To observe differences in survival between the virus-infected groups and the DEX-CYP control group, *P* values were calculated for all groups in comparison with the DEX-CYP control group (treatment, no infection). The null hypothesis states that both groups are not showing significant differences in survival. For this experiment, the observed differences in survival are all significant (p-value <0.05) except for the PUUV strain CG1820, implying that there is a difference in survival between the virus-infected groups and the DEX-CYP control group. *P* values were respectively calculated with the Log-rank test and Gehan-Breslow-Wilcoxon test for each virus group: BAYV, CDV, BCCV, ANDV and SNV, Log-rank: *p* value <0.0001, Gehan-Breslow-Wilcoxon: *p* value = 0.0002; LNV and MAPV, Log-rank: *p* value = 0.0251, Gehan-Breslow-Wilcoxon: p value = 0.0265; and CHOV; Log-rank: p value = 0.0024, Gehan-Breslow-Wilcoxon: p value = 0.0032.

### Infection of immunosuppressed hamsters with BAYV and BCCV mimics HPS

Three groups of 20 female golden Syrian hamsters were challenged with BCCV, BAYV and sterile phosphate-buffered saline. Hamsters were treated with DEX/CYP as described in Table 1. In the groups challenged with BCCV and BAYV all animals died within 21 days after infection. The animals from the control group challenged with sterile phosphate-buffered saline all survived the experiment and were still alive 35 days post infection. Two animals of each group were sacrificed on day 7, 10, 12, 15 and 18 days post infection. Liver, lungs and kidneys were removed and further investigated for signs of disease / histological changes and the presence of hantavirus RNA. RT-PCR performed on kidney, liver and lungs from the infected groups showed the presence of viral RNA in all animals.

**Fig 2:**
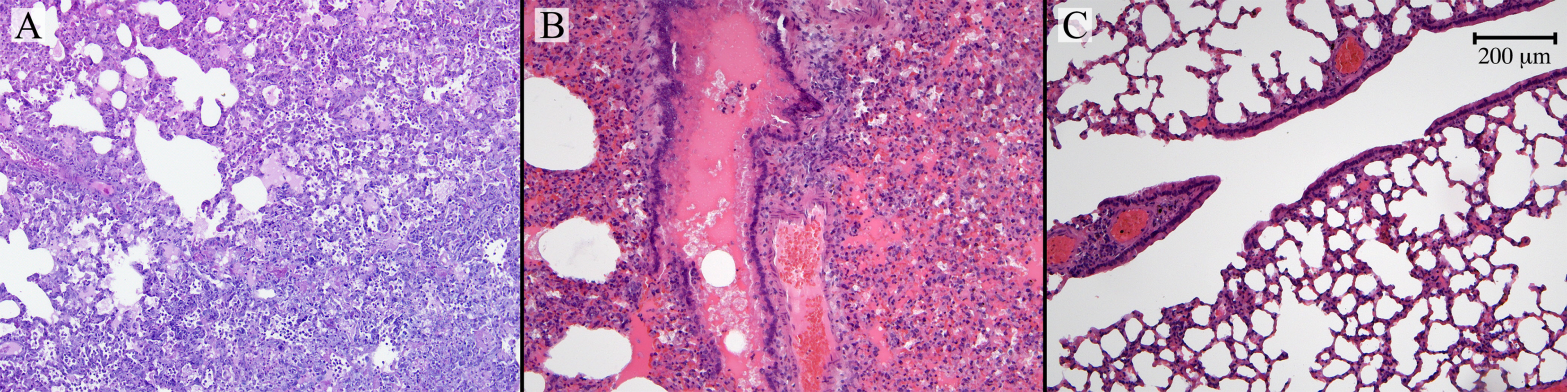
Histology of lung tissue from BAYV-, BCCV- and uninfected immunosuppressed hamsters. A) Lung biopsy showing a diffuse acute inflammation. Alveoli are filled with edema, mostly polymorphic inflammatory cells and fibrin (H&E x 100; Black Creek Canal virus group). B) Lung biopsy showing severe hemorrhagic edema in the alveoli, spilling over in the bronchiolar lumen (H&E x 100; Bayou virus group) C) Lung biopsy showing normal alveolar parenchyma and bronchioles (H&E x 100; Dex-Cyp control group).

Hematoxylin and eosin staining (H&E) was used to check the lung tissue for pulmonary abnormalities. Fig 2 shows the lung tissue taken from 3 different animals from different experimental groups. Animals infected with BCCV (Fig 2A) and BAYV (Fig 2B) exhibit a diffuse acute inflammation: alveoli are filled with edema, polymorphic inflammatory cells and fibrin. A severe hemorrhagic edema in the alveoli, spilling over in the bronchiolar lumen can also be observed (Fig 2B). The treated control group had normal lungs with no observed abnormalities in the parenchyma and bronchioles (Fig 2C).

### Passive immunization

**Fig 3:**
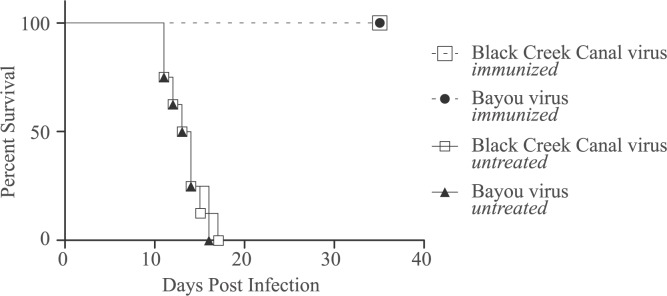
Survival curve of the passive immunization. Two groups, BAYV and BCCV, were both immunized with respectively 10,000 units of BAYV- or BCCV-antibodies 1 day prior to infection with 2,000 PFU of BAYV or BCCV. Two control groups, BAYV untreated and BCCV untreated, were not immunized but only infected with 2,000 PFU of BAYV or BCCV. The immunized groups were all alive at the end of the experiment on day 35 post infection. Both control groups showed a 100% mortality rate between 11 and 17 days post infection.

Passive immunization was carried out to confirm that the virus was responsible for the observed disease and mortality. For this experiment 4 groups of 8 hamsters were treated with dexamethasone and cyclophosphamide according to the dosing scheme explained in Table 1. Two test groups received 10,000 neutralizing antibody units of BCCV or BAYV on day -1. On day 0 all groups were infected with 2,000 PFU of BCCV. In the control groups animals died between 11 and 17 days post infection. In the test groups, receiving the BAYV and BCCV antibodies, all 8 hamsters in each group were still alive at the end of the experiment on day 35 post infection (Fig 3).

## Discussion

Several attempts have been made to establish an animal model for hantavirus-like diseases that reflects a human-like disease [21]. The first real breakthrough was the development of a lethal animal model for Andes virus in Syrian hamsters by Hooper et al [13]. Their findings paved the way for further research on this model and the possibility to further adapt it, as was done for Sin Nombre virus using immunosuppressive agents [14]. In our current study we tested several New World hantaviruses using an adapted immunosuppressive dosing scheme based on what was done before with the SNV model [14]. In a first experiment mortality was induced in all the infected New World hantaviruses groups with mortality rates going up to 100% for Black Creek Canal virus, Bayou virus, Caño Delgadito virus, Sin Nombre virus and Andes virus. For Laguna Negra virus, Maporal viru and Choclo virus mortality rates were noted ranging from 50 to 75%, respectively.

A previous study tested the pathogenicity of CHOV in immunocompetent Syrian golden hamsters and saw no mortality [22]. It was demonstrated that infection of endothelial lung cells alone does not result in mortality. In addition no edema in the lungs could be seen leading to the conclusion that the cellular inflammatory response might play an important role in the hamster pathogenesis of an HPS-like disease after infection with ANDV or MAPV [22]. The establishment of several New World hantavirus lineages over time, which are associated with molecular changes potentially altering disease severity and immune regulation, could harbor a possible explanation for the differences seen in pathogenicity [22].

Recently a hamster model using Turkish hamsters was described for LNV. In this model 5 different hamster species were infected with SNV, ANDV, MAPV and LNV. Only when LNV was used for infection in Turkish hamsters mortality was observed (43%) [23]. In the immunocompetent Syrian hamster model only ANDV and MAPV have shown to cause lethality, indicating that not only the virus is responsible for hantavirus pathogenesis [13, 24]. These findings seem to suggest a specific interaction between virus and host. In this light, studying the preferential binding of different virus strains to specific cellular receptors can be an interesting path for further research.

The fact that immunosuppressive agents are able to make Syrian hamsters vulnerable for hantaviruses other than Andes virus indicates that an interesting and probably complicated interplay between the virus and the immune system is responsible for the occurrence of hantavirus disease and its associated mortality. Even more interesting is the question what differentiates Andes virus from other New World hantaviruses, since they have high phylogenetic similarity. Currently, several hypotheses have been proposed to explain the observed discrepancies in pathogenicity between the different hantavirus strains. Thus far, Andes virus is the only known strain that gives 100% mortality in immunocompetent hamsters [13]. In a recent study it was shown that the nucleocapsid protein of ANDV inhibits RIG-I/MDA5-mediated IFN signaling, unlike the N protein of other New World hantaviruses [25]. This signaling pathway is normally triggered by the presence of double stranded RNA in the cytoplasm. The presence of cytoplasmic dsRNA is detected by RIG-I and MDA5, which are a class of pattern-recognition receptors (PRRs) [26]. In the case of ANDV there is a reduced IFN-I response, a pathway that has been demonstrated to play a central role in the innate hantavirus antiviral response [27]. These RIG-I/MDA5 findings were in line with previous research about the regulation of cytoplasmic helicase-directed IFN signaling by the ANDV N protein [25, 28, 29]. Additionally, a study on Hantaan virus was able to show an interaction between the N protein and the IFN-ß response depending on the amounts of N protein present [30]. Andes N protein by itself is able to activate specific signaling pathways (Rheb and RhoA GTPases) that control the size and permeability of microvascular endothelial cells [31]. Another explanation for the increased mortality of ANDV could be the necessity of the sterol regulatory pathway in the course of an ANDV infection. Manipulation of cholesterol levels has an effect on ANDV entry, suggesting that controlling sterol synthesis could have an antiviral effect. Furthermore, methyl-b-cyclodextrin, which reduces cellular cholesterol, induces a 10-fold inhibition on infection with ANDV [32, 33]. The impact of altering cholesterol levels on the function of T-cells as well as the influence of fatty acids and LDL on the functions of T-cells has been described [34–36]. The interplay of lipids and cholesterol and the function of T-cells is an interesting path to further explore for hantavirus infections as well as other viral infections by members of the order *Bunyavirales*. The role of lipid concentrations in the cell and plasma membrane could be of importance for the establishment of viral infections as well as viral entry when mediated through raft-dependent endocytosis [37, 38].

Besides ANDV there are other examples of hantavirus strains showing a different infection potential depending on the genotype. Dobrava-Belgrade (DOBV) is an Old World hantavirus causing hemorrhagic fever with renal syndrome in Europe, with case fatality rates going up to 15% depending on the genotype [39]. Differences in gene expression between the DOBV genotypes Dobrava, Sochi and Kurkino have been studied. Between these genotypes several variations were observed in the regulation of immune response genes and especially interesting are the differences in the expression of type-I interferon signaling [39].

Histology of selected tissues showed a clear difference between the control group and the virus-infected groups. The control group received immunosuppression but did not show any signs of disease. This indicates that the observed histological findings in the virus-infected groups are the result of the viral infection and not due to the treatment. This was also supported by the passive immunization, which clearly indicated that the virus is responsible for the signs of disease.

The observed clinical signs and onset of disease are alike with what has been described for HPS in humans and the ANDV model in Syrian hamsters. This is remarkable since it has been suggested that hantavirus pathogenesis is the result of an overactive immune response, which is being suppressed here by dexamethasone and cyclophosphamide treatment.

Future research should focus more on understanding the mechanisms of disease after infection with specific hantaviruses to find effective countermeasures. Why are some virus strains more lethal and how to they induce mortality? How do they interact with the immune system and how is this related to disease development?

Several New World virus strains were able to induce mortality up to 100% indicating that disease and mortality can be induced by using immune suppression. For the Old World viruses this was not seen, indicating that the viral aspect in the disease development is an important feature. It has been suggested several times that the immune system or the modulation of the immune system could be responsible for disease development. WBC counts in the SNV immunosuppressed model showed a 3- to 4-fold reduction compared to the control group indicating that the adaptive immune response is significantly depleted by a combination of cyclophosphamide and dexamethasone [14]. This indicates that the adaptive immune response is impaired, suggesting it may play an essential role in disease development. Contrary to these findings it was seen that depletion of CD4^+^ and CD8^+^ T cells in Syrian hamster before infection did not lead to lethality when infecting with ANDV [40, 41].

A good working animal model is a necessity for the development of good antiviral products. Several deadly New World hantaviruses have been identified as the causative agent of HPS. A broad-spectrum antiviral compound active against several hantaviruses would be the best outcome from an economic and scientific point of view. Novel antivirals are being tested, some showing promising results but *in vivo* testing is limited to the ANDV model or animal models lacking the human-like disease [10, 42]. The use of this robust immunosuppressed model, allowing the testing of antivirals against several New World hantaviruses, is presenting a solution to overcome this problem.

## Supporting information

S1 Table Mean weights of the different hamster groups tested at the start and end of the experiment. Corresponding p-values were calculated for each of the groups. For hamster groups with a 100% mortality rate a significant weight loss was observed.
(DOCX)
