# Supplementary material for: A lethal disease model for New World hantaviruses using immunosuppressed Syrian hamsters
Source: PLoS Negl Trop Dis. 2017 Oct 27;11(10):e0006042. doi: 10.1371/journal.pntd.0006042 (PMC5678717; doi:10.1371/journal.pntd.0006042)
Supplement: S1 Table — Corresponding p-values were calculated for each of the groups. For hamster groups with a 100% mortality rate a significant weight loss was observed. (DOCX) [file pntd.0006042.s001.docx]

|  | **Starting weights** | | **Endpoint weights** | |  |  |
| --- | --- | --- | --- | --- | --- | --- |
|  | *mean* | *range* | *mean* | *range* | *p-value* | *Mortality* |
| **DEX/CYP** | 130.8 | 124-140 | 135.8 | 129-147 | 0.3467 | 0% |
| **PUUV** | 137.0 | 134-143 | 124.5 | 120-136 | 0.0776 | 0% |
| **ANDV** | 142.5 | 135-152 | 115 | 106-123 | ***0.0007*** | 100% |
| **SNV** | 131.3 | 127-132 | 108.8 | 105-112 | ***0.0001*** | 100% |
| **BAYV** | 128.3 | 117-139 | 102.3 | 95-109 | ***0.0012*** | 100% |
| **BCCV** | 134.3 | 129-138 | 112 | 95-120 | ***0.0350*** | 100% |
| **LNV** | 121.0 | 119-126 | 110 | 103-125 | 0.0503 | 50% |
| **CDV** | 120.3 | 116-123 | 89.5 | 79-97 | ***0.0024*** | 100% |
| **CHOV** | 135.5 | 123-148 | 126 | 119-135 | 0.1364 | 75% |
| **MAPV** | 144.3 | 136-152 | 134.5 | 107-154 | 0.3668 | 50% |
|  |  |  |  |  |  |  |
